# Cardiovascular magnetic resonance findings in patients with *PRKAG2* gene mutations

**DOI:** 10.1186/s12968-015-0192-3

**Published:** 2015-10-24

**Authors:** Pauli Pöyhönen, Anita Hiippala, Laura Ollila, Touko Kaasalainen, Helena Hänninen, Tiina Heliö, Jonna Tallila, Catalina Vasilescu, Sari Kivistö, Tiina Ojala, Miia Holmström

**Affiliations:** Heart and Lung Center, University of Helsinki and Helsinki University Hospital, Po BOX 340, Helsinki, 00029 HUCH Finland; Children’s Hospital, University of Helsinki and Helsinki University Hospital, Helsinki, Finland; HUS Medical Imaging Center, Radiology, University of Helsinki and Helsinki University Hospital, Helsinki, Finland; HUS Medical Imaging Center, Clinical Physiology and Nuclear Medicine, University of Helsinki and Helsinki University Hospital, Helsinki, Finland; Blueprint Genetics, Helsinki, Finland; Molecular Neurology Research Program, Biomedicum Helsinki, University of Helsinki, Helsinki, Finland

## Abstract

**Background:**

Autosomal dominantly inherited PRKAG2 cardiac syndrome is due to a unique defect of the cardiac cell metabolism and has a distinctive histopathology with excess intracellular glycogen, and prognosis different from sarcomeric hypertrophic cardiomyopathy. We aimed to define the distinct characteristics of PRKAG2 using cardiovascular magnetic resonance (CMR).

**Methods:**

CMR (1.5 T) and genetic testing were performed in two families harboring *PRKAG2* mutations. On CMR, segmental analysis of left ventricular (LV) hypertrophy (LVH), function, native T1 mapping, and late gadolinium enhancement (LGE) were performed.

**Results:**

Six individuals (median age 23 years, range 16–48; two females) had a *PRKAG2* mutation: five with an R302Q mutation (family 1), and one with a novel H344P mutation (family 2). Three of six mutation carriers had LV mass above age and gender limits (203 g/m2, 157 g/m2 and 68 g/m2) and others (with R302Q mutation) normal LV masses. All mutation carriers had LVH in at least one segment, with the median maximal wall thickness of 13 mm (range 11–37 mm). Two R302Q mutation carriers with markedly increased LV mass (203 g/m2 and 157 g/m2) showed a diffuse pattern of hypertrophy but predominantly in the interventricular septum, while other mutation carriers exhibited a non-symmetric mid-infero-lateral pattern of hypertrophy. In family 1, the mutation negative male had a mean T1 value of 963 ms, three males with the R302Q mutation, LVH and no LGE a mean value of 918 ± 11 ms, and the oldest male with the R302Q mutation, extensive hypertrophy and LGE a mean value of 973 ms. Of six mutations carriers, two with advanced disease had LGE with 11 and 22 % enhancement of total LV volume.

**Conclusions:**

PRKAG2 cardiac syndrome may present with eccentric distribution of LVH, involving focal mid-infero-lateral pattern in the early disease stage, and more diffuse pattern but focusing on interventricular septum in advanced cases. In patients at earlier stages of disease, without LGE, T1 values may be reduced, while in the advanced disease stage T1 mapping may result in higher values caused by fibrosis. CMR is a valuable tool in detecting diffuse and focal myocardial abnormalities in PRKAG2 cardiomyopathy.

## Background

PRKAG2 cardiac syndrome is an autosomal dominantly inherited metabolic heart muscle disease characterized by left ventricular hypertrophy (LVH), progressive conducting abnormalities and ventricular pre-excitation (Wolff-Parkinson-White [WPW] syndrome) [1–4]. The prevalence is approximately 0.23–1 % in patients with hypertrophic cardiomyopathy (HCM) [4, 5]. Despite of the rarity, PRKAG2 accounts for a greater proportion of HCM in children and adolescents [6], and patients may benefit from early identification due to high risk of complete atrioventricular block [4, 7] and sudden cardiac death caused by atrial fibrillation and rapid antegrade conduction through an accessory pathway [8].

Patients with a *PRKAG2* gene defect have a distinctive cardiac histopathology with excess intracellular vacuoles filled with glycogen, myocyte enlargement but without myocyte disarray typical for sarcomeric HCM [3]. However, there are relatively few histopathological reports on PRKAG2 syndrome hearts, and in some cases abundant interstitial fibrosis and myocyte disarray in the absence of glycogen accumulation has been reported [4, 9]. Recently, severe fibrofatty myocardial replacement has been described in a patient with end-stage PRKAG2 [10].

In HCM, standard cardiovascular magnetic resonance (CMR) with late gadolinium enhancement (LGE) technique enables accurate assessment of myocardial morphology and function. While LGE visualizes the expansion of extracellular space related to focal myocardial fibrosis or infiltration [11, 12], T1 mapping has become a promising tool for evaluation of diffuse myocardial disease. Native T1 mapping reflects both intra- and extracellular signal in the myocardium [13]. Elevated native T1 times have been reported in fibrosis [14], edema [15] and amyloidosis [16], and lowered in iron overload [17] and focal fat infiltration [18]. Recently, native T1 mapping showed potential in the identification of Anderson-Fabry disease, a genetic storage disease characterized by multiorgan involvement and sphingolipid accumulation within myocytes [18, 19]. Metabolic defect resulting in macromolecular intracellular glycogen, and even lipid accumulation, may reduce native T1-relaxation time [20, 21], in the absence of significant extracellular fibrosis which is often seen in other forms of HCM.

To our knowledge, two case reports of CMR findings in PRKAG2 cardiomyopathy have been reported [22, 23]. As PRKAG2 cardiac syndrome is a unique glycogen storage cardiomyopathy, we sought whether CMR might demonstrate different imaging characteristics from sarcomeric HCM or hypertensive heart disease.

## Methods

### Patient population

This study was performed at the Helsinki University Central Hospital. All participants gave written informed consent and the project was approved by the local institutional ethics committee. Seven subjects from two separate families harboring *PRKAG2* mutations were recruited to this study. Two of the patients had a pacemaker system [24].

Clinical evaluation of all study subjects comprised review of hospital records, 12-lead electrocardiography (ECG) and echocardiography (index patients). Patients underwent electrophysiological study based on the treating cardiologist’s decision.

The phenotypic triad of PRKAG2 cardiac syndrome was based on observed cardiac hypertrophy, pre-excitation and conduction system disease [25]. Ventricular pre-excitation was defined as a short PR interval (<120 ms), widened QRS interval (>110 ms), and an abnormal initial QRS vector (delta-wave), or demonstration of an antegradely conducting accessory pathway on electrophysiologic study. Evidence of supraventricular tachycardia in association with pre-excitation defined WPW [25]. Diagnosis of conduction system disease required sinus node dysfunction or atrioventricular block on ECG.

### Genetic analysis

A novel next generation sequencing (NGS) strategy, oligonucleotide-selective sequencing [26], targeting 69 known genes associated with cardiomyopathies (Core Cardiomyopathy Panel, Blueprint Genetics, Helsinki, Finland, http://blueprintgenetics.com/), was used to examine the index patient in family 1. Median sequence coverage per base pair was 137x and 97.3 % of the target region was covered with >15x. Sequence analysis identified a heterozygous missense variant in the *PRKAG2* gene, c.905G > A (p.R302Q), which has been well characterized by several scientific reports as a disease mutation associated with dominantly inherited WPW and severe LVH resembling clinically HCM [2, 7, 9]. Direct Sanger sequencing was used to confirm the detected variant and family member testing.

The index patient in family 2 was screened using a custom-designed panel of 117 cardiomyopathy-related genes (HaloPlex kit of 500Kb, Agilent Technologies) and a heterozygous missense mutation was identified in the *PRKAG2* gene. The novel mutation p.H344P (c.1031A > C) was shown to segregate with the disease in the family and was absent in 3250 Finnish controls (Sequencing Initiative Suomi (SISu) database, http://www.sisuproject.fi/). The p.H344P mutation alters an evolutionarily conserved site in the protein, indicating that this amino acid presents an important role for the structure and the function of the protein. The variant was rated as deleterious and possibly damaging by the bioinformatics prediction tools SIFT and PolyPhen-2 respectively, strengthening the idea that this is a pathogenic variant.

### CMR technique

CMR was performed with 1.5 T MR scanner (Avantofit; Siemens, Erlangen, Germany) using a 32-channel receiver cardiac coil. Breath-hold cine MR was performed using retrospectively electrocardiographically gated segmented true fast imaging with balanced steady-state free precession (bSSFP) TrueFISP sequence. To assess left (LV) and right (RV) ventricular volumes and ejection fractions (EF) cine MR images were obtained in vertical, and horizontal long-axis, and a stack of short-axis planes covering both ventricles. Three-chamber view was obtained to assess LV outflow tract. The typical imaging parameters were TR/TE 3.0/1.6 ms, flip angle 52°, 256 × 256 matrix, 240 × 340 mm field of view (FOV). Slice thickness was 6 mm and interslice gap 20 %. The temporal resolution was 42–49 ms.

Myocardial T1 mapping was performed in a mid-ventricular short-axis slice using a shortened Modified Look-Locker Inversion-recovery (ShMOLLI) sequence. Typical acquisition parameters for ShMOLLI sequence were TR/TE 2.1/1.1 ms, flip angle 35°, 236 × 256 matrix and 331 × 360 mm FOV, inversion times varying from 90 ms to circa 5000 ms, and 8 mm slice thickness.

Quantitative myocardial T2 mapping was performed in a breath-hold fashion by using a T2-prepared bSSFP sequence to produce three single-shot T2-weighted images, each with different T2-preparation times (TE_T2P_ = 0 ms, 25 ms and 55 ms). The other scanning parameters were as follows: TR = 4 × RR intervals, flip angle 35°, 192 × 154 matrix, 360 × 289 mm FOV, and 8 mm slice thickness.

Ten minutes after injection of a contrast agent (gadoteratemeglumine, Dotarem® 0.2 mmol/kg) LGE images were acquired in the same views as for cine images, using inversion recovery spoiled gradient echo (IR-SPGR) sequence. The imaging parameters were TR/TE 2.58/2.3 ms, flip angle 50°, 256 × 256 matrix, 240 × 340 FOV. Slice thickness was 8 mm and interslice gap 0 %. Inversion time was optimized for each measurement to null the signal intensity of normal myocardium (240–360 ms).

Imaging of two patients with a permanent pacemaker device were performed according to previously published MR safety protocol for the pacemaker patients [24].

### Image analysis

Images were analyzed in consensus with two experienced (>10 years of experience) cardiac radiologists (SK, MH). In each patient, the three LV short axis sections were divided into 17 segments according to American Heart Association (AHA) guidelines [27]. Segments with any artifacts due to a pacemaker were excluded in all analysis.

Volumes, mass and wall thickness were assessed using standard protocols [28]. Imaging analysis was performed using QMass MR software® (version 7.6, Medis Medical Imaging Systems, Leiden, Netherlands). Cardiac hypertrophy was defined as increased LV wall thickness in one or more myocardial segments, or RV free wall thickness, of more than two standard deviations above the population mean (z-score > 2) on CMR.

Motion corrected T1 maps were generated and T1 estimates were computed on a per-pixel basis by performing a non-linear curve fitting using the three parameter signal model. We performed “midwall myocardial” segmental T1 analysis by measuring the mean T1-relaxation values separately for the anterior, anteroseptal, inferoseptal, inferior, inferolateral and anterolateral segments of myocardium (six segments). In addition to segmental analysis we also calculated the mean T1 value of the mid-myocardium. T1 values were compared with published values for normal healthy myocardium [29].

Acquired T2 images were motion corrected to in-plane motion between images by using a fast non-rigid registration algorithm. A pixel-wise myocardial T2 maps were generated using curve-fitting based on two parameter equation by assuming mono-exponential signal decay. Segmental analysis was performed for myocardial T2 values similarly as for T1 values.

The LGE percentage of LV was calculated using QMass MR software®. Hyperenhanced pixels were defined using full width at half maximum method.

### Statistical analysis

Continuous variables are presented as median (range) or mean ± standard deviation (SD) as appropriate, and categorical variables as frequency (%), unless otherwise mentioned. Comparison between continuous normally distributed variables was performed with Student’s *t*-test.

Results are given as absolute values and standardized to age, gender, body surface area (BSA, Mosteller’s method) and normal myocardial function (z-scores). Normal ventricular volumes and masses for adults were obtained from the references [28, 30] and for children (<18 years) from the reference [31], using similar 1.5 T MR scanner and bSSFP pulse sequence as ours.

Mean and maximal wall thickness of each of the 16 LV segments (apex left out) are presented as absolute (mm) and standardized (z-scores) values, with the aim to estimate the real distribution of segmental hypertrophy in relation to normal changes in wall thickness through the myocardium. Normal LV wall thickness values for adults were obtained from the reference [32] (similar scanner, sequence and QMass software tool as ours). Maximal absolute and standardized wall thickness values do not always represent the same segment.

Currently, there are no available reference values for LV wall thickness based on the 17-segment-model for children. Therefore, we used adult CMR references for the 17-year-old female (BSA 1.83 m2) and for the 16-year-old male (BSA 1.33 m2). BSA-standardized echocardiographic reference values were also used for all children for measurement of maximal wall thickness [33].

To take into account clustering and correlation of data within patients and myocardial segments, the association between segmental T1 values and hypertrophy were analysed using a linear mixed effects model, with patients treated as a random intercept and segments having a fixed effect on T1.

A *p*-value of < 0.05 was considered statistically significant and all tests were 2-sided. Statistical analysis was performed using the SPSS 21 package (SPSS, Chigaco, IL, USA). R language and environment for statistical computing (Version 2.15.3, R Core Team 2013, R Foundation for Statistical Computing, Vienna, Austria) was used for graphical output of segmental LV wall thickness and linear mixed effects model analysis.

## Results

### Study patients

**Fig. 1:**
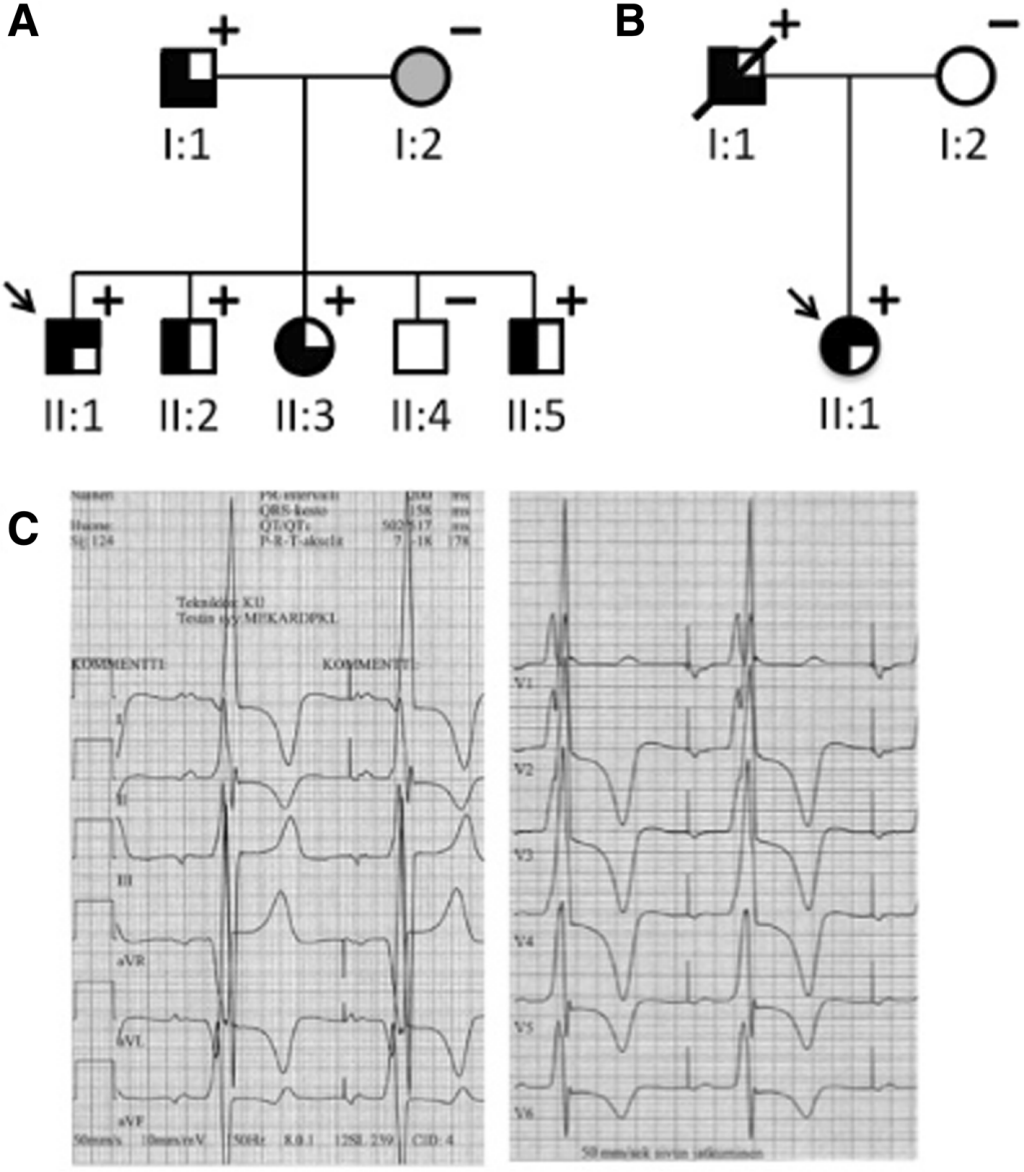
Pedigrees of two families with PRKAG2 cardiac syndrome and consequent clinical phenotypes of patients. Individuals with a *PRKAG2* mutation (+) were identified, with R302Q substitution in family 1 (**a**) and H344P substitution in family 2 (**b**). Squares indicate males and circles females. Filled symbols indicate disease phenotype in affected individuals, i.e., cardiac hypertrophy (left half filled), pre-excitation (right upper quadrant filled), or conduction system disease (right lower quadrant filled). Open symbols denote unaffected individuals and shading denotes uncertain clinical status. Arrows indicate index patients. The electrocardiogram of a patient with the R302Q mutation (**c**). Atrial pacing with large QRS deflections and pathologic T waves

Of the seven individuals examined with CMR in two separate families (Fig. 1a and b), altogether six had a *PRKAG2* mutation: five R302Q mutations in family 1 and one novel H344P mutation in family 2. The median age of the six mutation carriers (two females) was 23 years (range 16–48 years) and BSA 1.76 m^2^ (range 1.33–1.88 m^2^). One 19-year-old male did not have a *PRKAG2* mutation. Six mutation carriers completed otherwise full CMR imaging protocol, but one patient missed T1 and T2 mapping.

Clinical features of examined family members are presented in Table 1, and an abnormal electrocardiogram of a patient with the R302Q mutation in Fig. 1c. Based on hospital records, no other risk factors for LVH such as hypertension or chronic kidney diseases were detected in patients examined with CMR.

**Table 1:** Clinical features of study individuals

	PRKAG2 mutation	Age at CMR (yrs)	Age at Dx (yrs)	Sex	Pre-excitation	WPW	Conduction system disease^a^	PM	EP study ± RF ablation	Syncope	Cardiac hypertrophy	Tx	Death	Blood pressure (systolic/diastolic) mmHg	NYHA functional class	Serum Creatinine (μmol/l)	Hemoglobin (g/l)	NT-proBNB (ng/l)
Family 1																		
I:1	R302Q	48	30	M	-	-	+	+	-	+	+	-	-	136/80	1	91	165	^b^
II:1	R302Q	26	8	M	+	+	-	-	+	+	+	-	-	113/69	1	83	153	31
II:2	R302Q	24	24	M	-	-	-	-	+	-	+	-	-	110/57	1	^b^	^b^	25
II:3	R302Q	23	5	F	-	-	+	+	-	-	+	-	-	127/56	1	63	115	1078
II:4	None	19		M	-	-	-	-	-	-	-	-	-	124/62	1	^b^	^b^	^b^
II:5	R302Q	16	16	M	-	-	-	-	-	-	+	-	-	115/63	1	^b^	^b^	^b^
Family 2																		
I:1	H344P	^c^	24	M	-	-	+	+	-	+	+	+	+	160/100	3	58	120	39
II:1	H344P	17	10	F	+	+	-	-	+	-	+	-	-	116/65	1	129	146	1330

Mutation nomenclature is based on GenBank accession NM_016203.3 (PRKAG2) with nucleotide one being the first nucleotide of the translation initiation codon ATG

*Abbreviations*: *CMR* cardiovascular magnetic resonance, *Dx* diagnosis, *EP* electrophysiologic, *F* female, *LVH* left ventricular hypertrophy, *M* male, *NT-proBNB* N-terminal of the prohormone brain natriuretic peptide, *NYHA* New York Heart Association, *PM* permanent pacemaker, *RF* radiofrequency, *Tx* cardiac transplantation, *WPW* Wolff-Parkinson-White syndrome

^a^Sinus node dysfunction or atrioventricular block on ECG

^b^Data not available

^c^Was not examined with CMR. Cardiac hypertrophy diagnosis was based on the explanted heart

### Ventricular volumes, function and hypertrophy

**Fig. 2:**
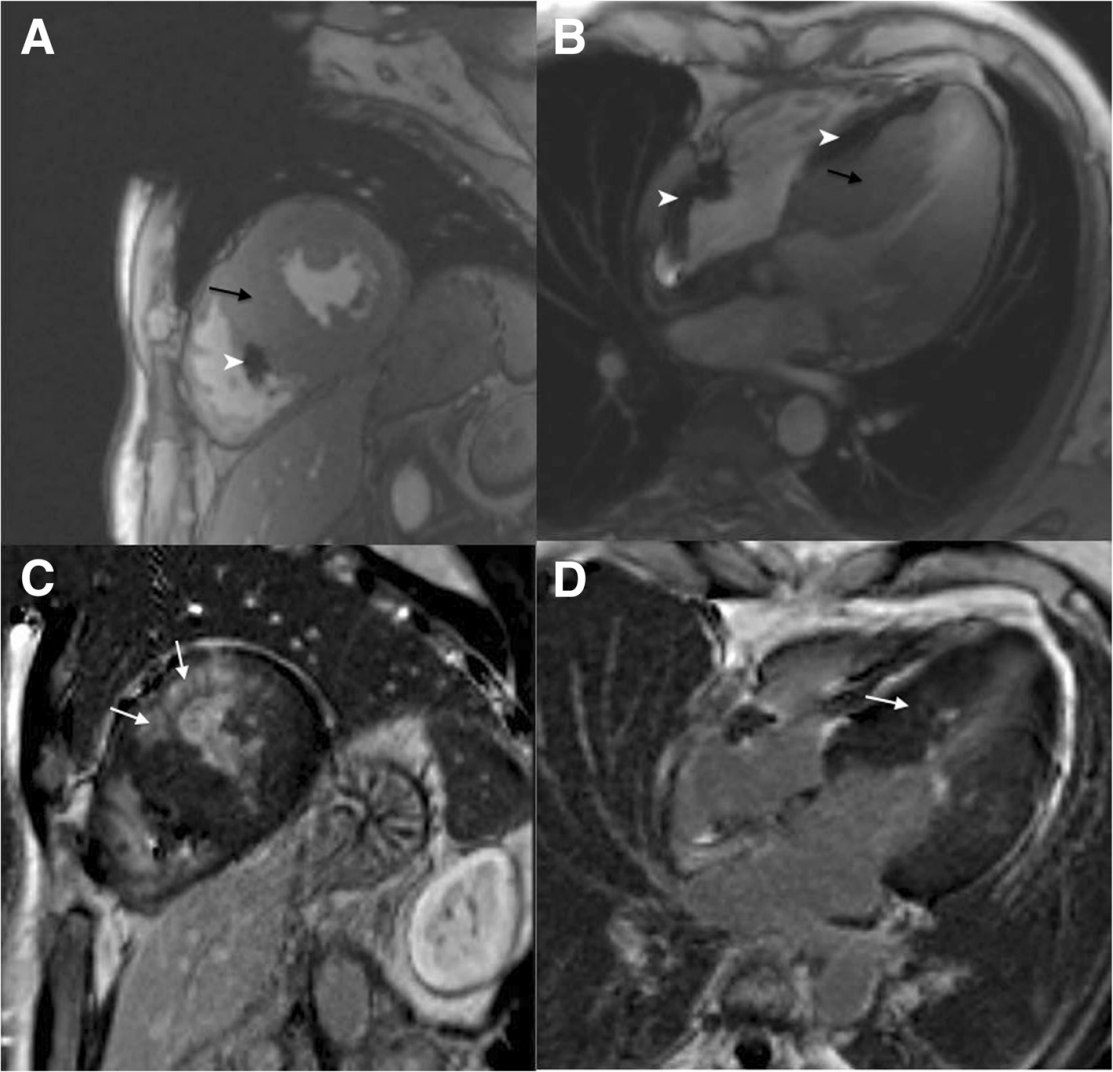
A 48-year-old male patient with PRKAG2 cardiac syndrome and a pacemaker. In short-axis and four chamber cine images septum is severely hypertrophied (**a**-**b**) (black arrows). Maximal septal and lateral wall thickness was 31 mm and 25 mm, respectively. Papillary muscles were excluded in the measurements. Anteroseptal, hypertrophic areas exhibit patchy late gadolinium enhancement (**c**-**d**) (white arrows). White arrow heads indicate artefact from pacemaker lead

**Fig. 3:**
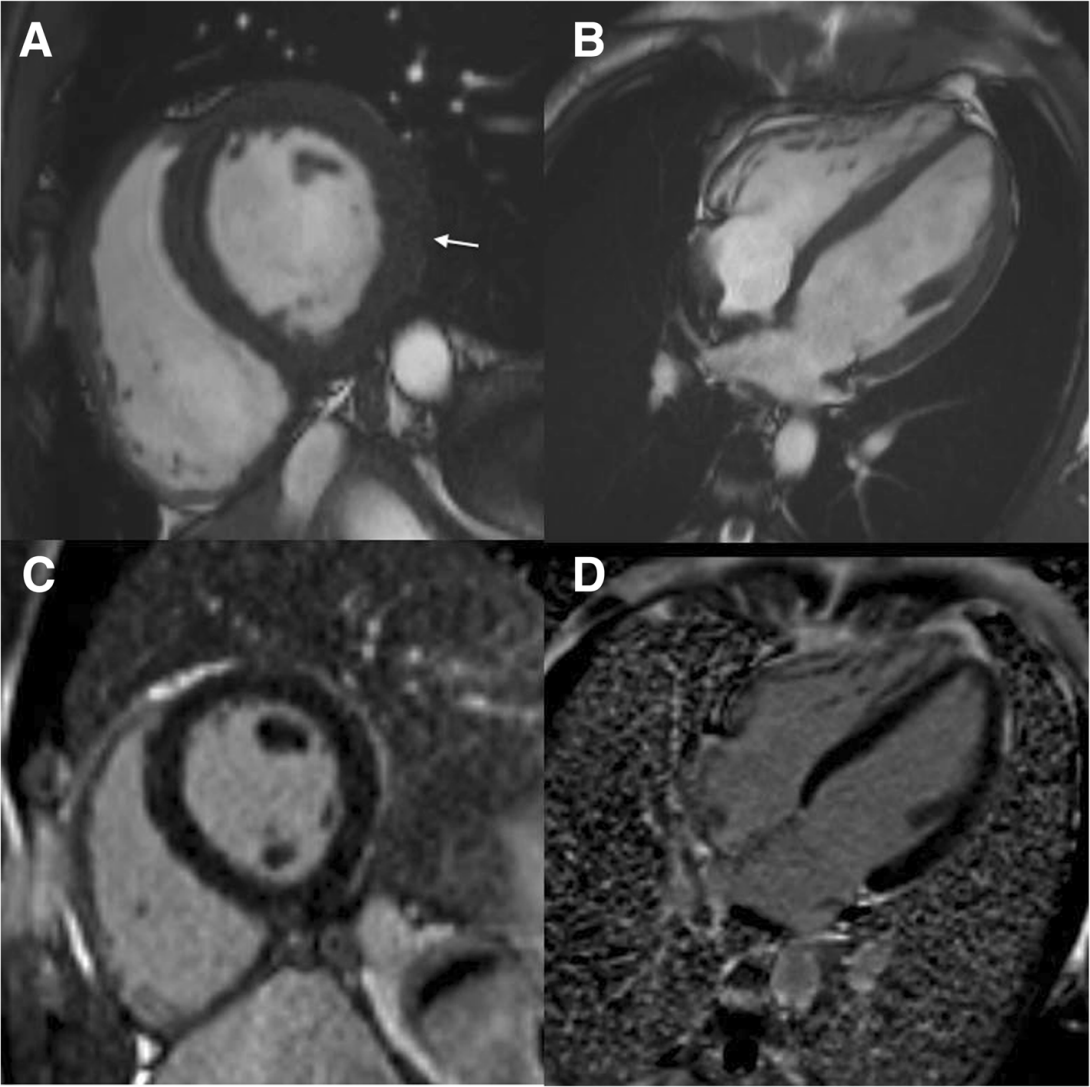
A 16-year-old male patient with a *PRKAG2* mutation. In short-axis cine image (**a-b**) inferolateral left ventricular wall is mildly hypertrophied (10–11 mm, maximal z-score 2.3) (white arrow), no late gadolinium enhancement is present (**c-d**)

All six *PRKAG2* mutation carriers had preserved age, gender and BSA standardized LV and RV end-diastolic volume (EDV), stroke volume and EF, see Table 2. However, three of six had LV mass above age and gender limits. In family 1, two R302Q mutation carriers (aged 23 and 48 years) had markedly elevated LV mass (203 g/m2 [z-score 18.8] and 157 g/m2 [9.6]) (Fig. 2) and three (aged 16, 24 and 26 years) normal LV masses (Fig. 3). In family 2, the 17-year-old female with an H344P mutation had also elevated LV mass (68 g/m2 [2.2]).

**Table 2:** Ventricular volumes, function and masses of *PRKAG2* mutation carriers compared to age, gender and body surface area standardized reference values

	All	
	(*n* = 6)	
	Value	z-score^a^
Left ventricle		
End-diastolic volume, ml	163 (116–180)	
End-diastolic volume index, ml/m2	90 (82–100)	0.6 (-0.1–1.6)
End-systolic volume, ml	63 (38–72)	
End-systolic volume index, ml/m2	34 (28–42)	0.9 (-1.0–2.2)
Stroke volume, ml	99 (78–109)	
Stroke volume index, ml/m2	57 (52–59)	0.3 (-0.3–1.0)
Cardiac output, l/min	5.9 (4.8–8.2)	
Cardiac output index, l/(min^a^m2)	3.5 (3.1–4.4)	
LVEF, %	62 (58–67)	−0.3 (-1.5–1.5)
Mass, g	137 (96–335)	
Mass index, g/m2	78 (68–203)	2.1 (-0.1–18.8)
Right ventricle		
End-diastolic volume, ml	167 (124–189)	
End-diastolic volume index, ml/m2	95 (75–102)	0.3 (-1.0–0.9)
End-systolic volume, ml	68 (38–83)	
End-systolic volume index, ml/m2	39 (23–45)	0.2 (-1.3–1.3)
Stroke volume, ml	99 (78–106)	
Stroke volume index, ml/m2	56 (52–59)	0.2 (-0.2–0.7)
Cardiac output, l/min	5.8 (4.9–8.2)	
Cardiac output index, l/(min^a^m2)	3.5 (3.1–4.4)	
RVEF, %	59 (56–69)	0.1 (-1.1–1.3)

Values are median (range)

*Abbreviations*: *LVEF* left ventricular ejection fraction, *RVEF* right ventricular ejection fraction

^a^Z-scores were calculated using the normal reference values for adults by Maceira A. et al. [30] and children by Buechel E. et al. [31]

**Fig. 4:**
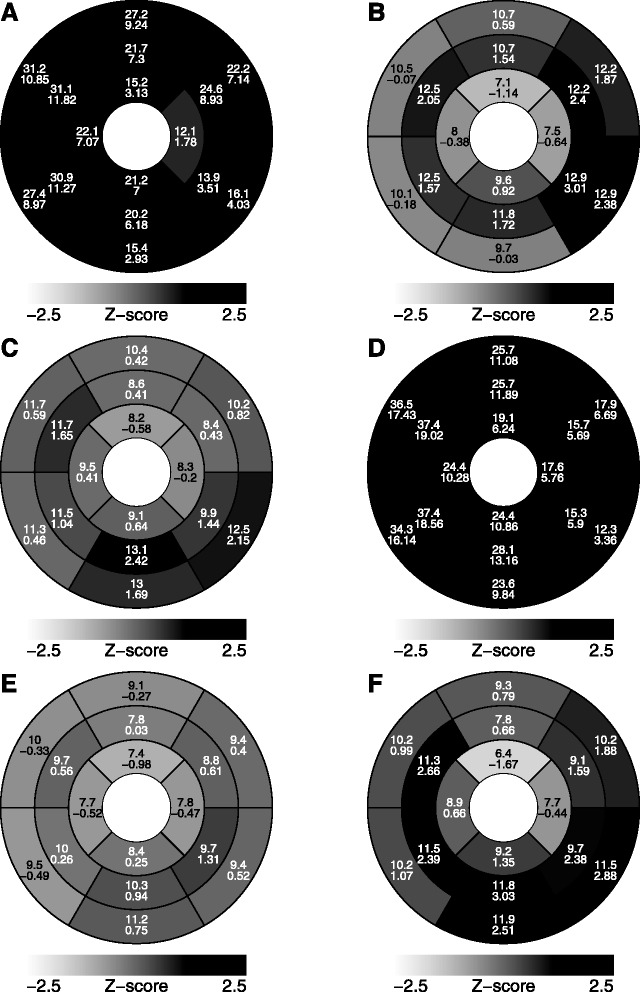
Distribution of left ventricular hypertrophy of six *PRKAG2* mutation carriers. Upper and lower values represent absolute (mm) and standardized (z-score) maximal wall thickness in the segment using adult references. Gray scaling of each segment is based on z-score. Members of family 1 with an R302Q mutation: I:1 (**a**), II:1 (**b**), II:2 (**c**), II:3 (**d**) and II:4 (**e**). A member of family 2 with an H344P-mutation: II:1 (**f**). The 16-year-old male (**e**), with body-surface-area (BSA) of 1.33 m2, had hypertrophy in mid-infero-lateral segment (posterior free wall) of 10 mm (z-score 2.3) using the BSA-standardized echocardiography based reference values for children

All mutation carriers had LVH by definition, i.e., wall thickness > 2 z-scores in one or more LV myocardial segments, with the median maximal wall thickness of 13 mm (range 11–37 mm) and z-score of 3.2 (range 2.3–19.0), see Table 3. Segmental distribution of maximal wall thickness had two different patterns (Fig. 4). Two symptomatic R302Q mutation carriers (aged 48 and 23 years) with markedly increased LV mass had hypertrophy throughout the myocardium, but predominantly in the interventricular septum (Fig. 4a and d), with the maximal (based on z-scores) wall thickness of 31 mm (z-score 11.8) and 37 mm (19.0) in the mid-anteroseptal segment in both patients. Trabeculae or papillary muscles were excluded in the measurements. Other mutation carriers with normal or only mildly increased LV mass (three R302Q carriers: aged 26, 24 and 16 years; one H344P carrier: aged 17 years) had LV hypertrophy in a non-symmetric pattern with the maximal wall thickness located in mid-inferolateral or mid-inferior segments (Fig. 4b, c, e and f).

**Table 3:** Summary of ventricular wall thickness compared to normal reference values and late gadolinium enhancement of *PRKAG2* mutation carriers

	All	
	(*n* = 6)	
	Value	z-score^a^
Left ventricle		
Left ventricular hypertrophy^b^	6 (100)	
Maximal wall thickness, mm	13 (11–37)	3.2 (2.3–19.0)
LGE presence	2 (33)	
LGE extent, % left ventricular volume^c^	17 (11–22)	
Right ventricle		
Free wall thickness, mm	5 (2–6)	
LGE presence	0 (0)	

Values are median (range) or n (%)

*Abbreviation*: *LGE* late gadolinium enhancement

^a^Standardized wall thickness (z-score), see Statistics section

^b^Left ventricular hypertrophy was defined as > 2 z-score wall thickening in one or more myocardial segments

^c^Only two patients had LGE

The median RV free wall thickness was 5 mm (range 2–6 mm).

### T1 and T2 mapping

In family 1, the mutation negative male (19 years old) had a mean T1 value of 963 ms, see Table 4. Three male siblings (26, 24 and 16 years) with the R302Q mutation, asymmetric LVH and without LGE, had a mean T1 value of 918 ± 11 ms. The oldest male in the family (48 years) with the R302Q mutation, extensive hypertrophy and LGE, had a mean T1 value of 973 ms (mid-anteroseptal, −anterior and -anterolateral segments excluded due to artifact caused by pacemaker), while corresponding segments had a mean LGE volume of 8 %.

**Table 4:** Mid-myocardium native T1 and T2 values of six study individuals (one R302Q mutation carrier lacked T1 and T2 mapping data)

	Segment						
	Anterior	Anteroseptal	Inferoseptal	Inferior	Inferolateral	Anterolateral	Mean
Native T1 values							
Family 1 (*n* = 5)							
R302Q mutation (*n* = 4)							
LGE negative (*n* = 3)							
1	934	932	944	905	897	929	924
2	914	902	925	924	879	894	906
3	922	938	945	919	905	918	925
Mean	923	924	938	916	894	914	918
LGE positive (*n* = 1)	m. v.	m. v.	990	944	986	m. v.	973
No mutation (*n* = 1)	984	954	938	960	984	955	963
Family 2 (*n* = 1)							
H344P mutation (*n* = 1)	950	965	985	968	957	969	966
Native T2 values							
Family 1 (*n* = 5)							
R302Q mutation (*n* = 4)							
LGE negative (*n* = 3)							
1	42.8	44.4	39.9	39.7	43.9	40.5	41.9
2	41.4	44.3	43.8	43.0	43.2	40.3	42.7
3	42.1	46.5	42.2	40.5	45.5	40.2	42.8
Mean	42.1	45.1	42.0	41.1	44.2	40.3	42.5
LGE positive (*n* = 1)	m. v.	m. v.	41.9	40.2	39.9	m. v.	40.7
No mutation (*n* = 1)	43.3	47.6	41.5	37.2	43.2	39.0	42.0
Family 2 (*n* = 1)							
H344P mutation (*n* = 1)	50.4	50.0	49.0	45.4	50.4	45.4	48.4

M.V. missing value (artefact by pacemaker)

*Abbreviation*: *LGE* late gadolinium enhancement

Considering 18 non-enhanced mid-myocardial segments in three male R302Q carriers, there was a trend toward significant inverse association between segmental T1 times and hypertrophy (segments with wall thickness z-score > 2 had a mean T1 value of 909 ± 16 ms versus other segments 925 ± 17 ms; *p* = 0.054). However, after adjustment to fixed effects caused by different segments on T1, the association between segmental T1 times and hypertrophy was non-significant (*p* = 0.194; linear mixed effects model).

In family 2, the female H344P mutation carrier (17 years) with asymmetric LVH and without LGE, had a mean mid-myocardium T1 value of 966 ms.

T2-relaxation times were within normal limits in all patients and there were no significant associations between hypertrophied segments and T2 time.

### LGE

Of the six mutations carriers, only two had left ventricular LGE, with 11 % and 22 % enhancement of total LV volume (Fig. 2). These patients also had severe LV hypertrophy. LGE was patchy and diffuse but focusing on the most hypertrophic segments. None of the patients presented LGE in the right ventricle.

### Histology

The father in family 2 (H344P carrier), who did not undergo CMR, had diffuse cardiac hypertrophy, LV and RV wall thickness ad 31 and 9 mm, intracellular vacuolization with positive periodic acid-Schiff (PAS) staining indicative of glycogen, and focal fibrosis in the explanted heart. There was also increased amount of left and right atrial fat.

### Mutation negative sibling

The 19-year-old mutation negative sibling in family 1 had normal LV mass and wall thickness, normal RV free wall thickness, but increased LV EDV (111 ml/m2 [2.8]) and decreased EF 55 % [−2.2].

## Discussion

We are the first to describe the comprehensive CMR findings of six individuals with *PRKAG2* mutation, known to cause a unique defect of the cardiac cell metabolism with intracellular deposition of glycogen. We found that the distribution of LVH in PRKAG2 syndrome is eccentric. Patients with markedly increased LV mass showed a diffuse pattern of hypertrophy but predominantly in the interventricular septum. On the other hand, four patients with normal or only mildly increased LV mass exhibited a non-symmetric mid-infero-lateral pattern of hypertrophy. T1 values were lowest in patients with the R302Q mutation, LVH and no LGE. Two patients with the *PRKAG2* mutation and advanced disease showed intramyocardial, patchy LGE in hypertrophic segments.

### Hypertrophy

We found that in five patients with the R302Q mutation (family 1), LVH demonstrated a similar diffuse pattern in two patients with markedly increased LV mass, but a non-symmetric mid-infero-lateral pattern in three patients with normal LV masses. Also, one patient with the H344P mutation (family 2) presented with non-symmetric mid-infero-lateral pattern of LVH. Significant LVH in PRKAG2 patients have been demonstrated previously by echocardiography [7, 34]. In the largest series of 45 *PRKAG2* mutation carriers, 78 % had LVH on echocardiography with a mean LV wall thickness of 21 mm (range 13–45 mm), and varying pattern of hypertrophy within families, 46 % having concentric, 29 % asymmetric, and one distal hypertrophy, with often eccentric distributions [4]. While concentric hypertrophy seems to be more common in metabolic and infiltrative cardiomyopathies [6], in sarcomeric HCM the distribution of LVH is characteristically asymmetric and heterogeneous, preferentially involving basal interventricular septum and often extending into the lateral wall, the posterior septum and LV apex [35].

Our finding of both diffuse and non-symmetric distribution of LVH in the same family may be explained by the generally progressive nature of LVH in PRKAG2 cardiomyopathy during follow-up [4], and the varying age of disease onset. That is, *PRKAG2* mutations may express in the early stage with focal and mild LVH, which may change to diffuse disease in the later stage. Another explanation could be, that even in the same family and with the same mutation, there would be varying patterns of hypertrophy. Nevertheless, our study demonstrates that CMR based accurate segmental analysis of wall thickness revealed abnormal hypertrophy in all *PRKAG2* mutation carriers, even in those subjects with normal LV mass.

### T1 and T2 mapping

Our study is the first one describing the behaviour of T1 or T2 times in a glycogen storage cardiomyopathy. In family 1, three males with the R302Q mutation, LVH but no LGE, presented with lower mean mid-myocardial T1 values (918 ± 11 ms) compared to mutation negative male sibling (963 ms) of their age and the mutation positive male with extensive hypertrophy and LGE (973 ms). This is in accordance with the hypothesized T1 reduction by intracellular glycogen in the absence of significant fibrosis. Theoretically, intracellular glycogen as a macromolecule with hydrophilic bonding with water would reduce T1-relaxation time, as shown in vitro [20, 21]. Possible lipid accumulation, demonstrated in a case report of end-stage PRKAG2, might also potent T1 shortening [10]. On the other hand, in the advanced disease stage T1 values may be slightly increased as a result of pseudonormalization caused by extracellular fibrosis, as seen in Anderson-Fabry disease [18, 19].

We were unable to verify the hypothesis of inverse association between segmental T1 times and hypertrophy in non-enhanced myocardium. After adjustment to fixed effects caused by different segments on T1, the association between T1 times and hypertrophy was non-significant. This may be due to limited number of patients and the fact that LVH was predominantly located in mid-infero-lateral segments which also present lower T1 values in normal myocardium [36].

In a previous study, it was estimated that normal native myocardial ShMOLLI T1 time is 962 ± 25 ms [29]. Piechnik et al. also showed that the principal biological parameter influencing myocardial ShMOLLI T1 is the female gender, T1 times of females under 45 years being 24 ms longer (blood 130 ms). In above 45-year-olds ShMOLLI T1 values did not differ between the sexes. The same study also reported no age-dependency of either myocardial or blood T1 values in males. In our very limited study population, the mean myocardial T1 value of the 17-year-old female H344P mutation carrier was 966 ms, whereas T1 values of male R302Q mutation carriers were from 906 to 973 ms; thus the differences caused by gender and mutation type may attenuate the possible changes related to intracellular glycogen.

All our patients had normal T2-relaxation times. Normal T2 times exclude possible iron accumulation in the myocardium and its effect on T1 values [37, 38].

### Late gadolinium enhancement

In this study, patients with significant LVH and increased LV mass, had widespread LGE. In an earlier case report of a patient with large cardiomyocyte vacuoles due to extensive cytosolic accumulation of glycogen in Danon disease (a mutation of the *LAMP2* gene), CMR demonstrated both RV and LV dysfunction, and extensive homogeneous LGE of the RV with concomitant patchy midwall LV involvement [39]. This was suggested to be caused by sarcolemmal damage in an advanced storage disease and subsequent gadolinium retention within intracellular space [39]. Similar mechanism of LGE has been suggested in Anderson-Fabry disease, with intracellular lipid accumulation [18], and could also explain LGE in our patients with the progressive stage of PRKAG2.

### Study limitations

The reference values used for calculating z-scores of LV wall thickness according to AHA 17-segment model were based on the largest available study on participants free of cardiac disease (similar software tool, MR scanner and sequence) with a mean age of 66 ± 9 years, which is considerably higher than our patients (median age 23 years, range 16–48). Therefore, we presented both absolute and standardized values of wall thickness when appropriate. In 16 myocardial segments, there is considerable probability to achieve abnormal z-scores in some segments by change. To overcome this, we plotted the distribution of z-scores in each patient to study the consistency of values in adjacent segments.

Our cohort was too small to find correlations between age, symptom duration and disease severity. We did not study the time-related changes in the myocardium. However, according to an earlier study, cardiac hypertrophy has a generally progressive nature in PRKAG2 [4], and thus different patterns of LVH in the same family may represent different stages of the disease. This study also lacks histological validation of imaging findings, although we presented the histology of the explanted heart of the father in family 2.

## Conclusions

PRKAG2 cardiac syndrome may present with eccentric distribution of LVH, involving focal mid-infero-lateral pattern in the early disease stage, and more diffuse pattern but focusing on interventricular septum in advanced cases. In patients at earlier stages of the disease, without LGE, T1 values may be reduced, while in the advanced disease stage T1 mapping may result in higher values caused by fibrosis. CMR is a valuable tool in detecting diffuse and focal myocardial abnormalities in PRKAG2 cardiomyopathy.

## Abbreviations

BSA: Body surface area
bSSFP: Balanced steady-state free precession
CMR: Cardiovascular magnetic resonance
ECG: Electrocardiography
EDV: End-diastolic volume
EF: Ejection fraction
FOV: Field of view
HCM: Hypertrophic cardiomyopathy
IR-SPGR: Inversion recovery spoiled gradient echo
LGE: Late gadolinium enhancement
LV: Left ventricular
LVH: Left ventricular hypertrophy
NGS: Next generation sequencing
PAS: Periodic acid-Schiff
RV: Right ventricular
ShMOLLI: Shortened Modified Look-Locker Inversion-recovery
WPW: Wolff-Parkinson-White
